# Recognizing the message and the messenger: biomimetic spectral analysis for robust speech and speaker recognition

**DOI:** 10.1007/s10772-012-9184-y

**Published:** 2012-12-18

**Authors:** Sridhar Krishna Nemala, Kailash Patil, Mounya Elhilali

**Affiliations:** Department of Electrical and Computer Engineering, Center for Language and Speech Processing, Johns Hopkins University, 3400 N Charles Street, Barton Hall, Rm 105, Baltimore, MD USA

**Keywords:** Multi-resolution, Speech recognition, Speaker verification, Biomimetic

## Abstract

Humans are quite adept at communicating in presence of noise. However most speech processing systems, like automatic speech and speaker recognition systems, suffer from a significant drop in performance when speech signals are corrupted with unseen background distortions. The proposed work explores the use of a biologically-motivated multi-resolution spectral analysis for speech representation. This approach focuses on the information-rich spectral attributes of speech and presents an intricate yet computationally-efficient analysis of the speech signal by careful choice of model parameters. Further, the approach takes advantage of an information-theoretic analysis of the message and speaker dominant regions in the speech signal, and defines feature representations to address two diverse tasks such as speech and speaker recognition. The proposed analysis surpasses the standard Mel-Frequency Cepstral Coefficients (MFCC), and its enhanced variants (via mean subtraction, variance normalization and time sequence filtering) and yields significant improvements over a state-of-the-art noise robust feature scheme, on both speech and speaker recognition tasks.

## Introduction

Despite the enormous advances in computing technology over the last few decades, progress in the fields of automatic speech recognition (ASR) and automatic speaker verification/recognition (ASV) still faces tremendous challenges when dealing with realistic acoustic environments and signal distortions. Tackling both speech and speaker feats adds additional hurdles since information about the speaker identity and the speech message tends to be reflected in slightly distinct yet overlapping components of the speech signal. For instance, whereas formant frequencies convey crucial information about the articulatory configuration of the vocal tract, they also reveal details about speaker-specific vocal tract geometries. Yet, our brains efficiently decode the signal information pertaining to *both* speech content and speaker identity using a common front-end machinery that is quite robust even at relatively high levels of distortion and noise (Greenberg et al. 2004).

Mel-Frequency Cepstral Coefficients (MFCC) are a classic example of the successful influence of biological intuition onto speech technologies, making them a staple in state-of-the-art ASR and ASV systems (Chen and Bilmes 2007; Kinnunen and Lib 2010). MFCCs provide a compact form of representing spectral details in the speech signal, that is motivated by both perceptual and computational considerations. They exploit the unique nature of frequency mapping in the auditory system, by warping the linear frequency axis into a nonlinear quasi-logarithmic scale. They also allow the decoupling of the speech production source and vocal tract characteristics via homomorphic filtering. In doing so, they highlight information about both the characteristics and configuration of the speech articulators that can be translated into a parametrization of both the identity of the speaker as well as the content of the speech message. While quite efficient and successful in conveying this information, features like MFCCs remain limited by their global analysis of the frequency spectrum. For instance, the first few coefficient describe details of the spectral tilt and compactness in the spectrum; but across *all* frequencies. Such broad analysis scatters information in specific frequency regions across all cepstrum coefficients.

In contrast, our knowledge of the central auditory system reveals that neurons in the auditory midbrain and primary auditory cortex exhibit a tuning to spectral details that is localized along the tonotopic axis (Schreiner and Calhoun 1995; Miller et al. 2002; Escabi and Read 2005). Such neural architecture provides a detailed multi-resolution analysis of the spectral sound profile that can bear great relevance to the front-end feature schemes used in speech and speaker recognition systems. Only few studies have attempted to translate the intricate multiscale cortical processing into algorithmic implementations for speech systems, yielding some improvements for ASR tasks (in noise) albeit at the expense of great computational complexity (Woojay and Juang 2007; Wu et al. 2009). To the best of our knowledge, no similar work was done for speaker recognition.

Admittedly, translating neurophysiological strategies into compact and efficient signal processing methods comes with a number of challenges; which have often hindered the introduction of biomimetic front-ends for such complex tasks as ASR or ASV (Stern 2011). They often amount to complex and computationally-intensive mappings that are impractical to use in real systems. In the present work, we set out to devise a simple, effective, and computationally-efficient multi-resolution representation of speech signals that builds on the principles of spectral analysis taking place in the central auditory system. By carefully optimizing the choice of model parameters, the analysis constrains the signal encoding to a perceptually-relevant subspace that maximizes recognition in presence of noise while maintaining computational efficiency. Further, unlike any of the previous approaches, speech (linguistic message) and speaker (identity) dominant regions in the signal encoding are analyzed, and different parameters are defined for speech and speaker recognition tasks. By employing the same front-end processing machinery, we maintain a generic framework for speech processing that can change parameters to shift focus either towards speech content information for ASR tasks or speaker information for ASV tasks. The following section describes details of the proposed multi-resolution spectral model and motivates the choice of its parameters. Next, we describe the experimental setup and results. We finish with a discussion of the proposed analysis, and comment on potential extensions towards achieving further noise robustness.

## The auditory multi-resolution spectral (AMRS) features

The parameterization of speech sounds is achieved through a multistage model that captures processing involved at different levels of the auditory pathway. All speech signals are first processed through a pre-emphasis stage, implemented as a first-order high pass filter with pre-emphasis coefficient 0.97. The one-dimensional acoustic signal *s*(*t*) are mapped onto a time-frequency representation referred to as auditory spectrogram, following an auditory-inspired model of cochlear and midbrain processing detailed in Lyon and Shamma (1996), Yang et al. (1992) and Wang and Shamma (1994).

**Fig. 1:**
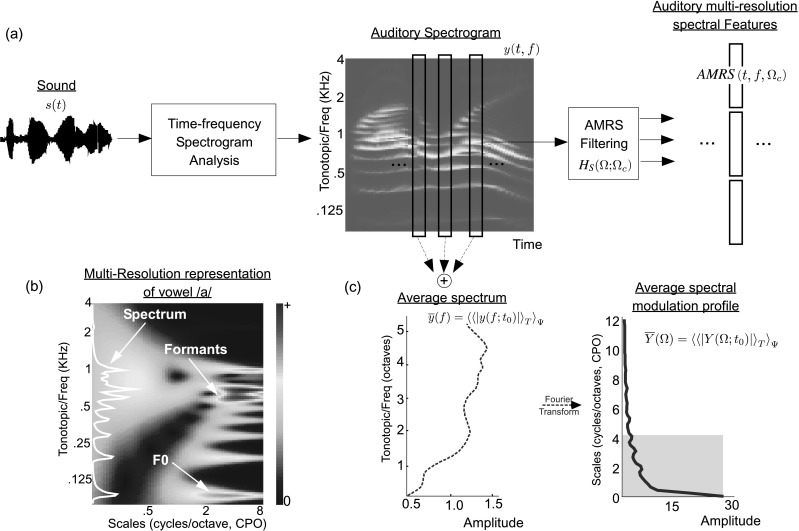
(**a**) Processing stages starting from an acoustic waveform *s*(*t*) to obtain AMRS features, parameterized by time *t*, tonotopic frequency *f* and spectral modulation filter parameter *Ω*_*c*_. (**b**) Example of spectral details revealed by AMRS analysis for vowel /a/ (**c**) (*left*) Average auditory spectrum computed over the TIMIT corpus, $\overline{y}(f)=\langle \langle \vert y(f;t_{0})\vert \rangle _{T}\rangle _{\varPsi}$; (*right*) Average spectral modulation profile, $\overline{Y}(\varOmega)=\langle \langle \vert Y(\varOmega;t_{0})\vert \rangle _{T}\rangle _{\varPsi}$

The first step consists of cochlear-filtering. This stage involves convolving the speech signal *s*(*t*) with a bank of 128 constant-*Q* (*Q*=4), highly asymmetric, bandpass filters *h*(*t*;*f*), equally spaced on a logarithmic frequency axis (Eq. (1a)). This operation results in a time-frequency cochlear spectrogram *y*_*coch*_(*t*,*f*). Next, a spectral sharpening operation takes place, by taking a first-difference over neighboring channels, followed by a half-wave rectification (Eq. (1b)). The loss of phase-locking at the level of the midbrain is then modeled by a short-term integration over 10 ms windows, followed by a cubic-root compression of the spectrogram (Eqs. (1c), (1d)). The outcome of this analysis is a transformation of the one-dimensional signal *s*(*t*) into a time-frequency spectrogram *y*(*t*,*f*) (Fig. 1(a)). The resultant spectrogram exhibits a number of characteristics; most importantly, in preserving detailed speech information such as formant structure as well as exhibiting noise robustness qualities over conventional representations (Shamma 1988; Byrne et al. 1989; Wang and Shamma 1994): 
1a
1b
1c
1d

The spectrogram reveals layered information about the speech signal that is distributed over different frequency bands and varying over multiple time-constants. The next stage of processing extracts detailed information about the spectral shape in *y*(*t*,*f*) via a bank of modulation filters operating in the Fourier domain resulting in the spectral cortical representation. The analysis mimics the spectral tuning of neurons in the central auditory pathway in which individual neurons are not only tuned to specific tonotopic frequencies (like cochlear filters); they are also selective to various spectral shapes, in particular to peaks of various widths on the frequency axis, hence expanding the cochlear one dimensional tonotopic axis onto a two-dimensional sheet (Schreiner and Calhoun 1995; Versnel et al. 1995). This analysis provides a more localized mapping of the spectral profile; that not only highlights details of bandwidth and spectral patterns in the signal but centers around the different frequency channels (Fig. 1(b)). Mathematically, the multi-resolution spectral analysis is modeled by taking the Fourier transform of each spectral slice *y*(*t*_0_,*f*) in the auditory spectrogram and multiplying it by a modulation filter *H*_*S*_(*Ω*;*Ω*_*c*_). The inverse Fourier transform then yields the modulation filtered version of the auditory spectrogram.^1^ The spectral modulation filter *H*_*S*_(*Ω*;*Ω*_*c*_) is defined as

2 $$ H_S(\varOmega;\varOmega_c) = (\varOmega/ \varOmega_c)^2 e^{ [1-(\varOmega/\varOmega_c)^2 ]} , \quad 0 \le \varOmega \le \varOmega_{\max}, $$

where *Ω* represents spectral modulations (or *scales*) and has units of cycles/octave (CPO), parameterizing the spectral resolution at which the auditory spectrogram is analyzed. *Ω*_max_ is the highest spectral modulation frequency set at 12 CPO (given the spectral resolution of 24 channels per octave).

### Choice of scales

There are two important aspects in defining the auditory multi-resolution spectral (AMRS) features for a specific task (ASR or ASV): (i) the span of the modulation filters; and (ii) the distribution of filters over the chosen span. In the current study, we constrain the range of scales to less than 4 CPO, since they cover more than 90 % of the entire spectral modulation energy in speech (Fig. 1(c)) and are shown to be most crucial for speech comprehension (Elliott and Theunissen 2009). To determine the filter distribution over the range 0–4 CPO, we employ a judicious sampling scheme in which the modulation regions with concentrated energy are sampled more densely; while the regions with less energy are sampled more coarsely. The set of scales *Ω*_*c*_ is chosen by dividing the average spectral modulation profile of speech (computed over the entire train data of TIMIT corpus (Garofolo et al. 1993)) into equal energy regions. The average spectral modulation profile $\overline{Y}(\varOmega)=\langle \langle \vert Y(\varOmega;t_{0})\vert \rangle _{T}\rangle _{\varPsi}$ is defined as the ensemble mean of the magnitude Fourier transform of the spectral slice *y*(*t*_0_,*f*) averaged over *t*_0_ and over all speech data *Ψ*. The resulting ensemble profile, shown in Fig. 1(b), is then divided into *M* equal energy regions *Γ*_*i*_:

3 $$ \varGamma_i = \int_{\varOmega_{i}}^{\varOmega_{i+1}} \overline{Y}(\varOmega)d\varOmega, \qquad \varGamma_i= \varGamma_{i+1}, \quad i=1,\ldots,M-1, $$

where *Ω*_*i*_ and *Ω*_*i*+1_ denote the lower and upper cutoffs for *k*th band, *Ω*_1_=0, and *Ω*_*M*_=4.

**Fig. 2:**
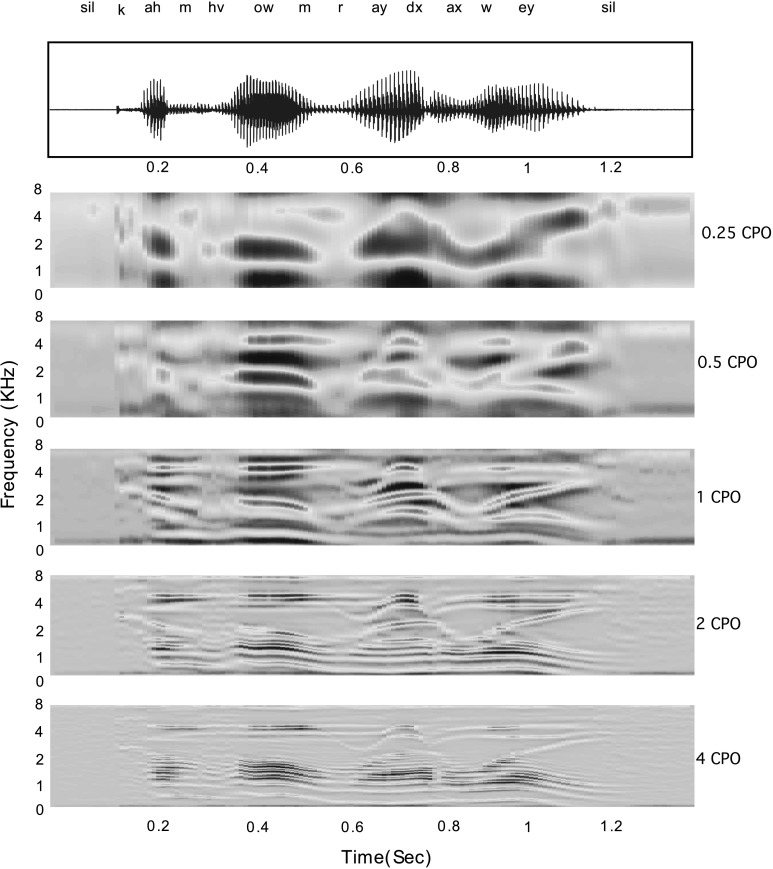
Illustration of the spectral modulation filtering at scales 0.25, 0.5, 1.0, 2.0, and 4.0 CPO for the utterance “*come home right away*” taken from TIMIT speech database. The *top panel* shows the time domain waveform along with the underlying phoneme label sequence

The scheme has the dual advantage of (i) implicitly encoding the high energy signal components which are inherently noise robust (ii) sampling the given modulation space with a smaller set of scales which is important both in terms of computation complexity as well the dimensionality of the resulting feature space. Setting *M*=5, the sampling scheme results approximately in a log-scale in the spectral modulation space, at 0.25, 0.5, 1.0, 2.0, and 4.0 CPO.^2^ The output of the five spectral modulation filters for an example speech utterance is shown in Fig. 2.

### Encoding of speech and/vs speaker information

The speech signal, discounting the environmental and channel effects, carries information about both the underlying linguistic message and the speaker identity (Fig. 1(b)). This information is manifested in slightly distinct yet overlapping components, and to separate these components is in general a non-trivial task. The spectral modulation filtering described above captures the overall spectral profile including formant peaks by employing broad scale filters (0.25 and 0.5 CPO) as well as narrower spectral details such as harmonic and subharmonic structures using higher resolution filters (1, 2 and 4 CPO). In order to select a set of scales (*Ω*_*c*_) that are relevant for diverse tasks such as speech and speaker recognition, we analyze the mutual information (MI) between the feature variables (*X*) encoding various scales and the corresponding (i) underlying linguistic message (*Y*_*l*_) (ii) speaker identity (*Y*_*s*_). The MI, a measure of the statistical dependence between random variables (Cover and Thomas 2006), is defined for two discrete random variables *X* and *Y* as:

4 $$ I(X;Y) = \sum_{x \in X,y \in Y} p(x,y) \log_2 \frac{p(x,y)}{p(x)p(y)}. $$

**Fig. 3:**
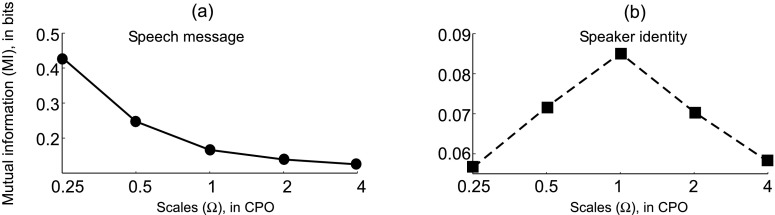
Mutual Information (MI) between feature representations encoding different scales and speech message (*left panel*), MI between feature representations encoding different scales and speaker information (*right panel*)

To estimate the MI, the continuous feature variables are quantized by dividing the range of observed features into cells of equal volume. To characterize the underlying linguistic message, phoneme labels from the TIMIT corpus are divided into four broad phoneme classes—the variable *Y*_*l*_ thus taking 4 discrete values representing the phoneme categories: vowels, stops, fricatives, and nasals. The average MI, taken as the average of the MI computed across all the frequency bands for any given scale, between the feature representations at different scales and the speech message is shown in Fig. 3(a). In the case of speaker identity, the ‘sa1’ speech utterance (*She had your dark suit in greasy wash water all year*) taken from the TIMIT corpus is compared across 100 different speakers—the variable *Y*_*s*_ taking 100 discrete values representing the speaker identity. The average MI between different scales and speaker information is shown in Fig. 3(b).^3^

Notice that the lower scales clearly provide significantly more information about the underlying linguistic message, while the speaker information is centered around 1 CPO—probably highlighting the significance of overall spectral profile including formant peaks in encoding speech message and the significance of pitch or harmonically-related frequency channels in representing speaker-specific information. In order to put more emphasis on message-dominant information present in the speech signal, it is important to encode information captured by lower scales for the speech recognition task. Consequently, for the speaker recognition task it is useful to encode information captured by higher scales. Therefore, in the feature encoding for the speech recognition task we choose *Ω*_*c*_={0.25,0.5,1.0,2.0} CPO and for the speaker recognition task *Ω*_*c*_={0.5,1.0,2.0,4.0} CPO.

Finally, the filtered spectrograms (one for each scale in *Ω*_*c*_) are downsampled in frequency by a factor of 4. This is achieved by integrating the 128 frequency channels into 32-bands, equally-spaced on a log-frequency axis.^4^ The final AMRS features are defined as 128 dimensional feature vector (32 auditory frequency channels multiplied by 4 scales) at each time frame of 10 ms. An estimate of processor usage shows that computing the multi-scale modulation filtering operation on top of the auditory-inspired spectrogram increases CPU time by about 75 % relative to an efficient implementation of Mel-Frequency Cesptral Coefficients.

## Experimental setup

### Phoneme recognition setup

Speaker independent phoneme recognition experiments are conducted on TIMIT database (excluding ‘sa’ dialect sentences), using the hybrid Hidden Markov Model/Multilayer perceptron (HMM/MLP) framework (Bourlard and Morgan 1994; Trentin and Gori 2003; Garcia-Moral et al. 2011). The training, cross-validation and test sets consist of 3400, 296 and 1344 utterances from 375, 87 and 168 speakers respectively. 61 hand-labeled symbols of the TIMIT training transcription are mapped to a standard set of 39 phonemes along with an additional garbage class (Lee and Hon 1989).^5^

MLP with a single hidden layer is trained to estimate the posterior probabilities of phonemes (conditioned on the input acoustic feature vector) by minimizing the cross entropy between the input feature vectors and the corresponding phoneme target classes (Richard and Lippmann 1991). Temporal context is captured by training a second MLP (in a hierarchical fashion) which operates on a longer temporal context of 23 frames of posterior probabilities estimated by the first MLP (Pinto et al. 2011). Both MLPs have a single hidden layer with sigmoid nonlinearity (1500 hidden nodes) and an output layer with softmax nonlinearity (40 output nodes). The final posterior probability estimates are converted to scaled likelihoods by dividing them with the corresponding prior probabilities (unigram language model) of phonemes. An HMM with 3 states, each with equal self and transition probabilities, is used for modeling each phoneme. The emission likelihood of its each state is set to be the scaled likelihood. Finally, the Viterbi algorithm is applied for decoding the phoneme sequence. Note that the hybrid HMM/MLP system achieves better phoneme recognition performance than the standard HMM/GMM systems (Garimella et al. 2010).

### Speaker recognition setup

Text independent speaker verification experiments using Gaussian Mixture Models (GMM) are conducted on a subset of the NIST 2008 speaker recognition evaluation (SRE) (NIST 2008). In our UBM-GMM based speaker recognition system (Kinnunen and Lib 2010), the Universal Background Model (UBM) is trained with data obtained from a set of 325 speakers. In the UBM training, a total of 256 mixtures and 10 expectation-maximization iterations for mixture split are used. A total of 85 target speaker models are obtained by *maximum a posteriori* (MAP) adaptation of the UBM. MIT Lincoln Lab GMM toolkit is used for the UBM-GMM training. An independent set of 500 test trials is used to evaluate the verification performance. The number of impostor and genuine trials in the test set are 169 and 331 respectively. The data represents training and testing from an interview setting using the same microphone (NIST 2008).^6^ This condition is specifically chosen in order to focus on additive noise distortions, without introducing other channel mismatch scenarios in the standard NIST SRE—hence ensuring consistency *across* ASR and ASV results in noise. Also, the UBM-GMM recognition backend does not include factor analysis techniques (Kinnunen and Lib 2010) which address various channel mismatch scenarios present in the NIST SREs. Notice however that the UBM-GMM system used even without the factor analysis techniques achieves state-of-the-art recognition performance on the same microphone matched channel condition evaluated in this work.

### Features

(i) For phoneme recognition experiments, each MFCC feature vector is obtained by stacking a set of 9 frames of standard 13 Mel frequency cepstral coefficients along with their first, second, and third order temporal derivatives.^7^ The AMRS feature vector is obtained by taking the original 128 dimensions (32 auditory frequency channels ×4 scales, as described in Sect. 2) along with their first, second, and third order temporal derivatives.

(ii) For speaker recognition experiments, each MFCC feature vector is obtained by taking 19 Mel frequency cepstral coefficients along with their first and second order temporal derivatives. Note that the higher order cepstral coefficients are more common in the speaker recognition literature and form the state-of-the-art feature representation in recent NIST SREs. Similarly, the AMRS feature vector is obtained by taking the base feature representation along with its first and second order temporal derivatives.

## Recognition results

### Performance of AMRS features

Extending the mutual information analysis presented in the Sect. 2.2, we empirically show the relevance of set of scales {0.25,0.5,1.0,2.0} CPO and {0.5,1.0,2.0,4.0} CPO for speech and speaker recognition tasks respectively. The performance of the AMRS features that encode these two sets of scales for the ASR and ASV tasks is shown in Table 1. Notice in particular how encoding the lower scales and omitting the higher scales improved the speech recognition performance, and vice-versa for speaker recognition task.

**Table 1:** Automatic speech recognition (ASR) and automatic speaker verification (ASV) performance of AMRS features. ASR performance is shown in phoneme recognition rate (PRR) and ASV performance is shown in equal error rate (EER)

Scales encoded in the features (CPO)	ASR performance (in PRR, %)	ASV performance (in EER, %)
[0.25,0.5,1,2]	**71.9**	3.4
[0.5,1,2,4]	68.7	**2.7**

### Comparison with standard front-end features

The proposed AMRS features are contrasted with MFCC features on both ASR and ASV tasks. To evaluate the noise robustness aspect of the two feature representations, various noisy versions of the test set are created by adding four types of noises at Signal-to-Noise-Ratio (SNR) levels of 20 dB, 15 dB, and 10 dB. The noise types chosen are, Factory floor noise (Factory1), Speech babble noise (Babble), Volvo car interior noise (Volvo), and F16 cockpit noise (F16), all taken from NOISEX-92 database, and added using the standard FaNT tool (Hirsch 2005). In all the experiments, the recognition models are trained only on the original clean training set and tested on the clean as well as noisy versions of test set (*mismatch* train and test conditions). The phoneme recognition accuracy and speaker verification performance of the MFCCs and the AMRS features is listed in Table 2. The proposed AMRS features achieve ASR and ASV performance comparable to that of MFCCs under clean conditions. With additive noise conditions reflecting a variety of real acoustic scenarios, the AMRS features perform substantially better than the MFCCs—an average relative improvement of 38.9 % on the ASR task and an average relative error rate reduction of 31.9 % on the ASV task.

**Table 2:** Automatic speech recognition (ASR) and automatic speaker verification (ASV) performance of MFCC and AMRSF feature representations for different types of noise

Noise type	SNR (in dB)	ASR performance (in PRR)		ASV performance (in EER)	
		MFCC	AMRSF	MFCC	AMRSF
Clean	∞	71.4	71.9	2.7	2.7
Factory1	20	48.2	61	7.1	5.9
	15	38.1	53.1	10.9	7.6
	10	28.3	42.7	17.8	11.4
	5	19.6	30.9	28.4	18.7
	Average	33.5	46.9	16.1	10.9
Babble	20	48.1	64.1	5.4	4.1
	15	37.3	55.8	7.9	5.9
	10	27.6	43.7	11.5	9.7
	5	19.5	29	24.8	14.2
	Average	33.1	48.1	12.4	8.4
Volvo	20	60.8	70.9	3.9	2.9
	15	55.7	70.7	4.6	3.4
	10	49.9	70.1	6.4	4.8
	5	42.9	68.9	10.9	6.5
	Average	52.3	70.1	6.4	4.4
F16	20	48.5	61.4	10.7	7.5
	15	37.8	53.3	16.3	10.7
	10	27	40.9	21.7	14.5
	5	18.2	27.2	29.9	21.1
	Average	32.8	45.7	19.6	13.4

### Comparison with state-of-the-art noise robust scheme

We further compare the performance of AMRS features with a state-of-the-art noise robust feature scheme, Mean-Variance ARMA (MVA) processing of MFCC features (Chen and Bilmes 2007). The MVA processing, when applied with the standard MFCC features, combines the advantages of multiple noise robustness schemes: cepstral mean subtraction, variance normalization, and temporal modulation filtering. The MVA has been shown to provide excellent robustness for additive noise distortions and form the state-of-the-art in noise robustness evaluations on the Aurora 2.0 and Aurora 3.0 databases (Chen and Bilmes 2007). Note that the auto-regression-moving-average (ARMA) filtering in the MVA processing is shown to be superior to temporal modulation filtering techniques like RASTA (Hermansky and Morgan 1994) for noise robustness.

To further improve the noise robustness of AMRS features and be consistent with the temporal modulation filtering employed in the MVA feature scheme, the AMRS features are processed with a bandpass modulation filter applied in the temporal domain.^8^ The filtering is done in the Fourier domain of the modulation amplitude. First the Fourier transform of the time sequence of each feature in the feature stream is taken, then is multiplied by a bandpass modulation filter *H*_*T*_(*w*;[0.5,12]) capturing the modulation content within the specified range of 0.5 Hz and 12 Hz. Note that this temporal modulation range has been shown to be *information rich* and crucial for speech comprehension (Elliott and Theunissen 2009). The inverse Fourier transform then yields the modulation filtered version of the feature stream. The bandpass modulation filter *H*_*T*_(*w*;[0.5,12]) is defined as follows: 
5 where *w*_max_ is the modulation frequency resolution—50 Hz corresponding to the 10 ms frame-rate of the feature stream.

The phoneme recognition accuracy and speaker verification performance of MVA and *enhanced* AMRS features (E_AMRSF) is shown in Table 3. In addition to being comparable in the clean/matched conditions, the E_AMRSF features perform significantly better than MVA features in noisy/mismatch conditions—an average relative improvement of 12.2 % on the ASR task and an average relative error rate reduction of 33.9 % on the ASV task.

**Table 3:** Automatic Speech Recognition (ASR) and Automatic Speaker Verification (ASV) performance of MFCC_MVA and E_AMRSF representations for different types of noise

Noise type	SNR (in dB)	ASR performance (in PRR)		ASV performance (in EER)	
		MFCC_MVA	E_AMRSF	MFCC_MVA	E_AMRSF
Clean	∞	68.2	69.5	3	2.9
Factory1	20	55.7	61.7	5.4	5.2
	15	48.4	55.3	10	6.5
	10	39.4	45.5	16.6	10.7
	5	30.2	34.3	23.9	16.3
	Average	43.4	49.2	13.9	9.6
Babble	20	56.5	64.5	4.5	3.9
	15	49.5	57.7	6.2	5.4
	10	40.7	48.1	10.7	8.9
	5	29.7	34.4	19.5	12.4
	Average	44.1	51.1	10.2	7.6
Volvo	20	63.5	69.4	3.6	3
	15	62	69.2	5.2	3.4
	10	60.2	68.6	6.5	4.6
	5	58.1	67.7	9.4	6.2
	Average	60.9	68.7	6.1	4.3
F16	20	57.1	61.8	12.4	7.3
	15	50.8	55.6	18.3	10.2
	10	43.2	46.4	22.4	12.4
	5	34.6	35.1	26.6	16.6
	Average	46.4	49.7	19.9	11.6

## Discussion

In this work, we begin to address the issue of versatile speech representations that could bear relevance to both speaker and speech recognition tasks. The proposed scheme captures the prominent features of the speech spectrum ranging from its broad trends (which correlate with vocal tract shape and length) to its rapidly varying details (which capture information about harmonics and voice quality). Because of the non-targeted nature of the proposed multi-resolution analysis, it is able to map the speech signal onto a rich space that highlights information about the glottal shape and movements as well as vocal tract geometry and articulatory configuration. Notice how the proposed analysis allowed for defining two slightly different feature representations for speech and speaker recognition tasks using the same feature analysis machinery. This multi-resolution representation can be viewed as a *local* variant (w.r.t log-frequency axis) of the analysis provided by the cepstral decomposition (MFCC). Spectral shape information in cepstral analysis is scattered over all cepstrum coefficients and hence must be considered collectively, and not individually. In the proposed localized approach, one can mine the information in each scale component individually. While the two methods perform comparably in clean, the proposed feature representations reveal substantial robustness under noisy conditions in both ASR and ASV tasks.

The current effort is not the first attempt at bringing more biological realism to analysis of speech signals. A number of authors have explored improvements to speech feature analysis that ranged from detailed modeling of the efferent auditory periphery, including intricate nonlinear effects and firing patterns at the auditory nerve (Seneff 1986; Beet and Gransden 1992; Ghitza 1994; Lee et al. 2011; Clark et al. 2012), cochleogram-type representations (Muthusamy et al. 1990), stabilized and normalized auditory image representations (Patterson et al. 2010), to even more selective model-based spectro-temporal fragments and dynamic maps (Brown et al. 2001; Barker et al. 2010). Auditory-inspired techniques have generally led to noticeable improvements over more ‘conventional’ signal processing methods for recognition tasks, particularly when dealing with distorted signals in presence of background or competing noises (Fanty et al. 1991; Jankowski and Lippmann 1992; Hermansky 1998). Additional techniques have also been proposed to take advantage of the multi-resolution scheme taking place at more central stations of the auditory pathway; whereby the spectral details of the signal as they evolve over time are meticulously analyzed via parallel channels that capture intricate details of the signal of interest. Recent implementations of such schemes have been shown to yield noticeable improvements to automatic speech recognition, particularly with regards to its noise-robustness (Woojay and Juang 2007). The current work falls in the same category of more centrally-inspired analysis of speech signals. It provides two major advantages over comparable methods (Woojay and Juang 2007; Wu et al. 2009): It does not involve dimension-expanded representations (close to 30,000 dimensions) which would inherently require tedious and computationally-expensive schemes hence limiting their applicability. Instead, our model is constrained to a perceptually-relevant spectral modulation subspace and further uses a judicious sampling scheme to encode the information with only four modulation filters. This results in a low-dimensional and highly robust feature space. The enhanced AMRS features also constrain temporal modulations to a perceptually-relevant space shown to be crucial for speech comprehension. Note that none of the components of the model have been calibrated to deal with a specific noise condition making it appropriate for testing in a wide range of acoustic environments.

Our ongoing efforts are aimed at achieving further improvements by applying the multi-resolution analysis on enhanced spectral profiles obtained from speech enhancement techniques (Loizou 2007) that benefit from additional voice/speech activity detectors and noise estimation/compensation techniques. Also, the noise robustness obtained here from AMRS features can extend to other large scale ASR tasks in TANDEM framework (Hermansky et al. 2000). Similarly, more elaborate ASV systems are achievable using AMRS features in conjunction with standard practices in speaker recognition like factor analysis, supervectors and score normalization (Kinnunen and Lib 2010).

## Footnotes

1 The modulation filtering is performed in real domain.

2 The original sampling results in spectral modulations at {0.18,0.59,1.34,2.36,4.0} CPO.

3 The difference in MI levels between the speech message and speaker identity may be attributed to the observation that the speech signal encodes more information about the underlying linguistic message as compared to speaker information.

4 This reduction of the spectral axis resolution did not affect the ASR/ASV performance.

5 It is possible to achieve higher recognition performance (in *clean* or *matched* condition) by using a larger set of 49 labels during the training and mapping to the standard set of 39 phonemes only during the scoring.

6 Corresponds to condition 2 of the eight common conditions evaluated in the NIST 2008 speaker recognition evaluation.

7 The 9 frame context window and the resulting 468 dimensional feature representation achieved best recognition performance, better than the standard 39 dimensional MFCC features (Nemala et al. 2011).

8 Note that the MVA processing has been shown to be *optimal* for cepstral domain features (Chen and Bilmes 2007). Consequently, it may be sub-optimal to apply the same processing on the AMRS features.
